# Rho-independent stimulation of axon outgrowth and activation of the ERK and Akt signaling pathways by C3 transferase in sensory neurons

**DOI:** 10.3389/fncel.2012.00043

**Published:** 2012-10-11

**Authors:** Maria Auer, Rüdiger Schweigreiter, Barbara Hausott, Sitthisak Thongrong, Markus Höltje, Ingo Just, Christine Bandtlow, Lars Klimaschewski

**Affiliations:** ^1^Division of Neuroanatomy, Innsbruck Medical UniversityInnsbruck, Austria; ^2^Division of Neurobiochemistry, Innsbruck Medical UniversityInnsbruck, Austria; ^3^Centre for Anatomy, Charité-University Medicine BerlinBerlin, Germany; ^4^Institute of Toxicology, Hannover Medical SchoolHannover, Germany

**Keywords:** dorsal root ganglia, axon regeneration, RhoA, exoenzyme

## Abstract

Peripheral nerve injury triggers the activation of RhoA in spinal motor and peripheral sensory neurons. RhoA activates a number of effector proteins including the Rho-associated kinase, ROCK, which targets the cytoskeleton and leads to inhibition of neurite outgrowth. Blockade of the Rho/ROCK pathway by pharmacological means improves axon regeneration after experimental injury. C3_bot_ transferase, an exoenzyme produced by *Clostridium botulinum*, inactivates RhoA by ADP-ribosylation. It has been successfully applied in experimental CNS lesions to facilitate axon regeneration. Up to now it was not investigated thoroughly whether C3_bot_ exerts positive effects on peripheral axon regeneration as well. In the present study, recombinant membrane permeable C3_bot_ produced a small, but significant, axon outgrowth effect on peripheral sensory neurons dissociated from adult dorsal root ganglia (DRG) of the rat. Neuronal overexpression of C3, however, did not enhance axonal growth. Moreover, transfection of plasmids encoding dominant negative RhoA or RhoA specific shRNAs failed to increase axonal growth. Furthermore, we show that the C3_bot_ mutant, C3_*E*174*Q*_, which lacks RhoA inhibitory activity, still stimulates axonal growth. When analyzing possible signaling mechanisms we found that extracellular signal-regulated kinase (ERK) and Akt are activated by C3_bot_ and ERK is induced by the C3_*E*174*Q*_ mutant. Upregulation of kinase activities by C3_bot_ occurs significantly faster than inactivation of RhoA indicating a RhoA-independent pathway of action by C3_bot_. The induction of ERK signaling by C3_bot_ was detected in embryonic hippocampal neurons, too. Taken together, although RhoA plays a central role for inhibition of axon outgrowth by myelin-derived inhibitors, it does not interfere with axonal growth of sensory neurons on a permissive substrate *in vitro*. C3_bot_ blocks neuronal RhoA activity, but its positive effects on axon elongation and branching appear to be mediated by Rho independent mechanisms involving activation of axon growth promoting ERK and Akt kinases.

## Introduction

Following a peripheral nerve lesion axon regeneration results in restoration of function, but long distance regeneration continues to remain a major challenge. To this end, axon elongation needs to be promoted over axon branching that leads to neuroma formation at the lesion site and misdirected regeneration into the distal nerve stump (Grosheva et al., 2008). Small GTPases have been suggested to play a key role in axon elongation and branching by acting as intrinsic modulators of axon growth, either as positive (Rac1, Cdc42) or negative (RhoA) regulators (for a recent review see Auer et al., 2011). Peripheral nerve injury triggers the activation of RhoA in spinal motor and peripheral sensory neurons (Hiraga et al., 2006; Cheng et al., 2008). RhoA primarily mediates neurite retraction (Wahl et al., 2000), whereas Rac1A and Cdc42 induce lamellipodia and filopodia formation (Kozma et al., 1997). Pharmacological inhibition of RhoA or expression of dominant negative RhoA results in neurite outgrowth of neuronal cell lines (Nishiki et al., 1990; Kranenburg et al., 1997; Albertinazzi et al., 1998; Sebok et al., 1999).

RhoA activates several effector proteins including the Rho-associated kinase, ROCK, which targets the actin, actomyosin, and microtubule cytoskeleton resulting in retraction of growth cones and blockade of neurite outgrowth (Govek et al., 2005; Mimura et al., 2006). Some of the Rho/ROCK inhibitors show beneficial effects in lesion models of the CNS and PNS. The cell permeable form of C3_bot_ (BA-210 or Cethrin®; McKerracher and Higuchi, 2006), an exoenzyme produced by *Clostridium botulinum* that inactivates RhoA by ADP-ribosylation (Aktories and Just, 2005), has been successfully applied in various central nervous system lesion paradigms to improve axon regeneration functionally and morphologically (Lehmann et al., 1999; Dergham et al., 2002; Fischer et al., 2004). Recently, Cethrin® has successfully completed a phase I/IIa clinical trial (Fehlings et al., 2011). Similarly, the ROCK inhibitor HA-1077 (Fasudil®) facilitates regeneration in the injured CNS (Dergham et al., 2002; Fournier et al., 2003) and in the lesioned sciatic nerve (Hiraga et al., 2006; Cheng et al., 2008). The two latter studies revealed that ROCK inhibition improves peripheral nerve regeneration by increasing axon numbers and amplitudes of distally evoked compound muscle action potentials. Moreover, in recent studies small peptides derived from C3_bot_ were shown to promote axon regeneration and motor recovery in the lesioned central and peripheral nervous system (Boato et al., 2010; Huelsenbeck et al., 2012).

Here, we provide evidence that interfering with RhoA by pharmacological inactivation, down-regulation, or by a dominant-negative approach does not promote axon outgrowth of peripheral sensory neurons obtained from adult dorsal root ganglia (DRG). Membrane permeable C3_bot_, however, does exert positive effects on axon elongation and branching, but these occur Rho-independently, presumably by activation of the neuronal extracellular signal-regulated kinase (ERK) and Akt signaling pathways.

## Results

### Upregulation of RhoA activity upon dissection of DRG and counteracting effect of neuronal growth factors

**Figure 1:**
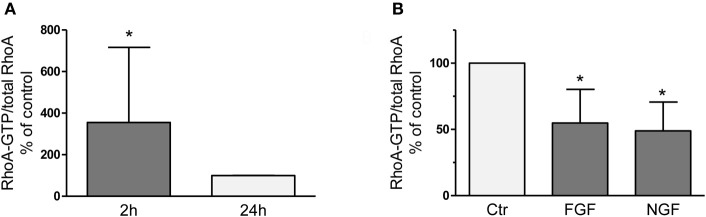
RhoA-GTP pull down assays performed 2 h or 24 h after dissociation and plating of adult DRG neurons on a growth promoting substrate **(A)**. Compared to the 24 h time point vehicle-treated naïve neurons reveal significantly increased RhoA-GTP levels after 2 h *in vitro*, which is counteracted by treatment with neuronal growth factors like FGF-2 or NGF (**B**; each 100 ng/ml for 2 h; *n* = 3, mean ± SD; ^*^p < 0.05).

RhoA-GTP pull down assays revealed 3-fold higher levels of active RhoA 2 h after dissection of adult sensory neurons as compared to 24 h after plating (Figure 1) corroborating activation of RhoA as observed recently in axotomized DRG *in vivo* (Hiraga et al., 2006; Cheng et al., 2008). We treated DRG cultures with neuronal growth factors FGF-2 or nerve growth factor (NGF; each 100 ng/ml for 2 h), because they are strongly induced at the lesion site, promote axon outgrowth (Hausott et al., 2009) and inhibit RhoA activity in a neuronal cell line (PC12; Nusser et al., 2002; Harada et al., 2005). We found that RhoA-GTP levels were decreased by 45 and 51%, respectively, suggesting that growth factor mediated inhibition of RhoA may contribute to improved axon regeneration. Consequently, we hypothesized that any other means to negatively interfere with RhoA-GTP loading could have beneficial effects on axonal growth as well. The RhoA inhibitor C3_bot_ is well known from a number of CNS studies to markedly promote regrowth of nerve fibers and functional recovery (McKerracher and Higuchi, 2006). Therefore, we applied C3_bot_ to dissociated adult DRG neuron cultures.

### Recombinant C3_bot_ stimulates axon outgrowth

**Figure 2:**
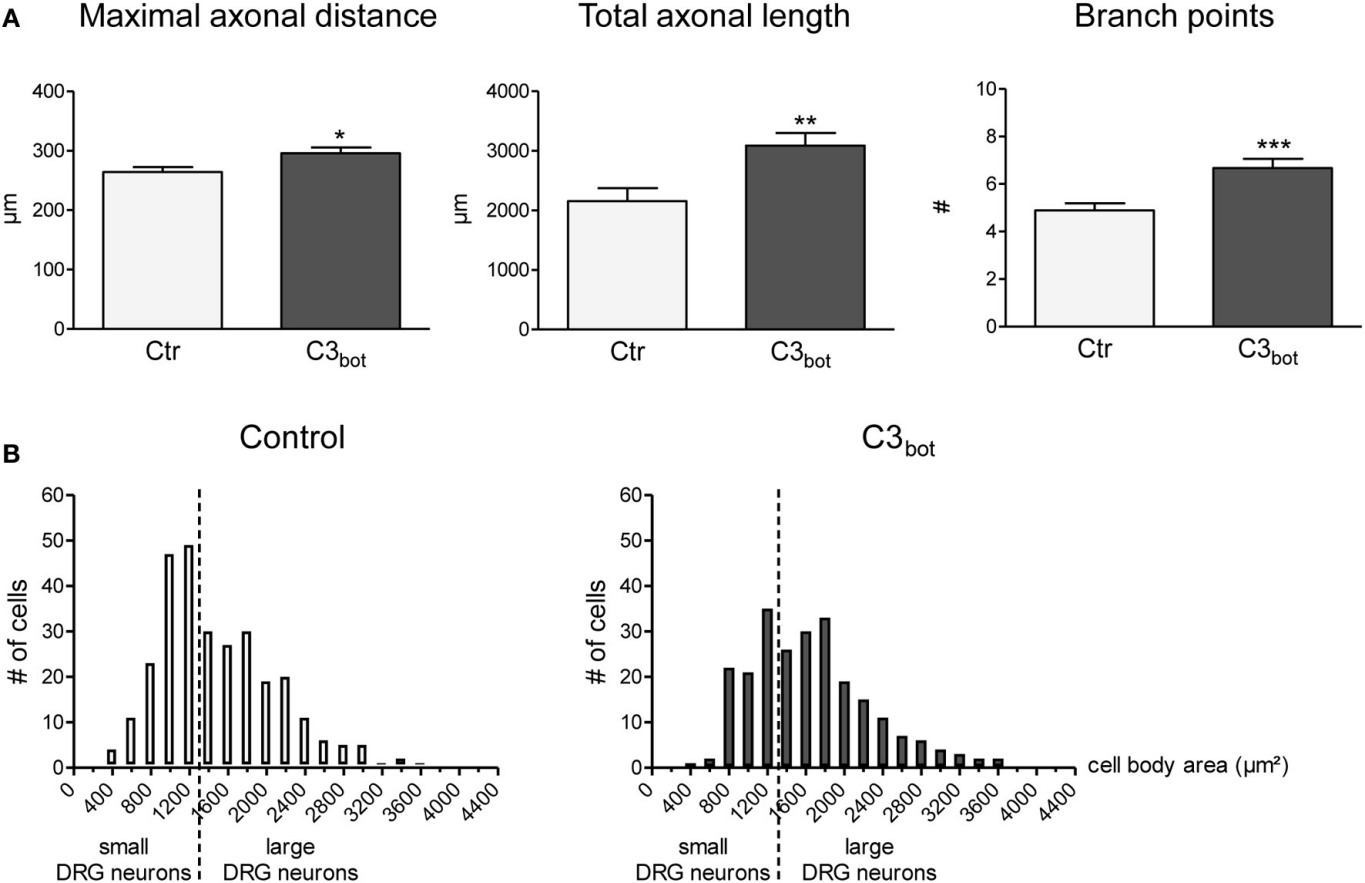
Application of the Rho inhibitor C3_bot_ (1 μg/ml, membrane permeable) for 24 h increases the length of the longest axon (maximal axonal distance), the extension of the axonal tree (total axonal length), and the number of branch points per neuron (**A**; total number of neurons per group >240, three independent experiments, mean ± SEM; ^*^p < 0.05, ^**^p < 0.01, ^***^p < 0.005). Histograms reflecting the size distribution of cultured rat DRG neurons **(B)**. DRG neurons with a cell body area spanning less than 1500 μ m^2^ are classified as small DRG neurons, those above 1500 μm^2^ as large DRG neurons (separated by a vertical line).

C3_bot_ treatment of sensory neurons derived from adult rat DRG for 24 h revealed a small, but statistically significant, positive axon outgrowth effect. The length of the longest axon (maximal axonal distance) increased by 12%, the total axonal length by 43% and the number of axonal branch points per cell was elevated by 36% (Figure 2A). Analogous to growth factor treatments (Yip et al., 1984), C3_bot_ enhanced neuronal soma size (Figure 2B). The mean area of vehicle-treated neuronal cell bodies (1551 μm^2^) was significantly smaller than of C3_bot_ treated cultures (1887 μm^2^) suggesting that C3_bot_ exerts a general trophic effect onto DRG neurons.

### Neuronal overexpression of C3_bot_ does not improve axon outgrowth

**Figure 3:**
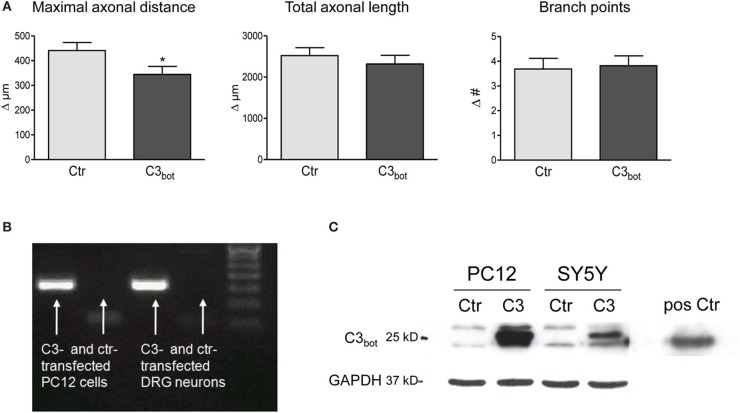
Neuronal transfection of a plasmid encoding for C3 (pEGF-C3) and a marker plasmid encoding DsRed does not stimulate axonal growth **(A)**. Axon growth between day 2 and day 3 (results presented as delta) after transfection is followed cell-by-cell. The maximal axonal distance of C3 overexpressing neurons decreases compared to control DsRed-transfected neurons. No alterations in the total axonal length and the number of branch points are observed (total number of neurons per group >110, three independent experiments, mean ± SEM; ^*^p < 0.05). Quantitative RT-PCR of transfected rat pheochromocytoma (PC12) cells and DRG neurons (20 cells per experiment) reveals C3 mRNA expression **(B)**. C3-transfected PC12 and SY5Y human neuroblastoma cells express high levels of C3 protein **(C)**. Pure C3_bot_ served as positive control.

After treatment of sensory neurons with recombinant C3_bot_ we hypothesized that neuronal overexpression of C3_bot_ would have an even stronger effect on axonal growth due to easier access to cytoplasmic RhoA. It was already known that C3_bot_ overexpression is sufficient to ADP-ribosylate and, thereby, inhibit RhoA in various cell lines and primary neurons (Bobak et al., 1997; Moorman et al., 1999; Semenova et al., 2007). Overexpression of pEGF-C3_bot_ in cultured sensory neurons, however, did not enhance axon growth (Figure 3A). The maximal axonal distance even decreased by 22%, whereas the total axonal length and the number of branch points were not altered in C3_bot_ transfected neurons. Grouped cell RT-PCR revealed strong C3_bot_ mRNA expression in C3 overexpressing neurons (Figure 3B). Due to the low transfection efficiency in primary neurons C3_bot_ protein levels were assessed in the two cell lines, PC12 and SY5Y. The protein was clearly detected in Western blotting experiments following overexpression of C3_bot_ (Figure 3C), which also induced neurite outgrowth in PC12 cell cultures (data not shown).

### Dominant negative RhoA mutants and shRNAs against RhoA have no effect on axon outgrowth

**Figure 4:**
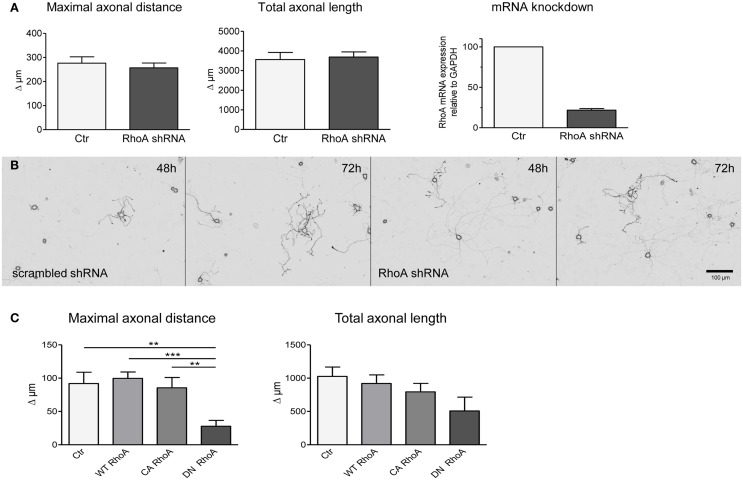
Transfection of shRNA plasmids (also encoding EGFP) specifically targeting RhoA does not alter axonal growth of DRG neurons between day 2 and day 3 (results presented as delta; **A,B**). Controls are transfected with respective scrambled shRNA (total number of neurons per group >60, three independent experiments, mean ± SEM). Quantitative RT-PCR reveals downregulation of RhoA mRNA in DRG neurons following RhoA shRNA transfection (pool of 20 neurons per PCR, *n* = 3). DRG neurons transfected with a plasmid encoding dominant negative (DN) RhoA together with a marker plasmid exhibit a decreased maximal axonal distance with a statistically unaltered total axonal length **(C)**. Constitutively active (CA) or wildtype RhoA do not alter axonal growth in fluorescent cells (total number of neurons per group >77, three independent experiments, mean ± SEM; ^**^p < 0.01, ^***^p < 0.005).

The lack of effect of neuronal C3_bot_ overexpression prompted us to interfere with neuronal RhoA expression directly. Four different shRNA sequences specifically targeting RhoA mRNA were analyzed in comparison to scrambled control shRNA plasmids. Down-regulation of RhoA mRNA levels by >80% did not enhance axon growth of DRG neurons measured at 2 and 3 days after transfection (Figures 4A,B). The efficacy of the silencing approach was confirmed by grouped cell qRT-PCR of DRG neurons (Figure 4A) and protein blotting of shRNA transfected PC12 cells (data not shown).

Subsequently, we overexpressed wild-type RhoA, constitutively active (V14) or dominant-negative (N19) RhoA constructs in DRG neurons. Transfection of plasmids encoding dominant negative RhoA did not enhance axon outgrowth, but, in fact, decreased the maximal axonal length in comparison to control transfected neurons (Figure 4C). Overexpression of wild-type or constitutively active RhoA did not show any effect on maximal axonal distance. None of the constructs had an impact on total axonal length, but V14 and N19 partly affected neuronal viability.

### The C3_bot_ mutant E174Q still promotes axonal outgrowth

**Figure 5:**
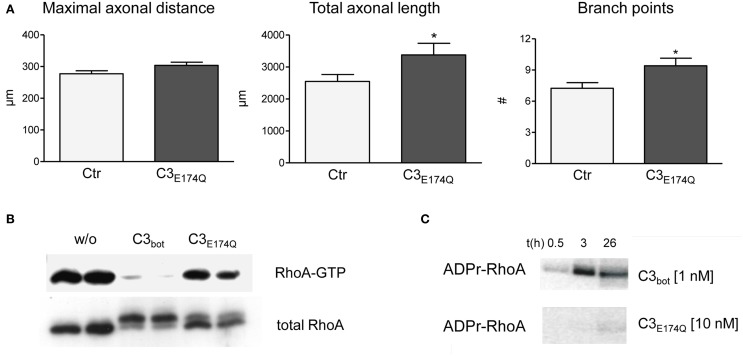
Compared to vehicle-treated control neurons mutant C3_*E*174*Q*_ (1 μg/ml, non-permeable) stimulates axonal growth, but does not improve axonal elongation (**A**; total number of neurons per group >240, three independent experiments, mean ± SEM; ^*^ p< 0.05). Pull down assays **(B)** demonstrate strongly reduced RhoA-GTP levels one day after treatment with C3_bot_, but only slightly with C3_*E*174*Q*_. A shift in molecular weight of total RhoA reveals modification by ADP-ribosylation. *In vitro* cell-free assays with radioactive ADP confirm inability of C3_*E*174*Q*_ to inactivate RhoA directly **(C)**.

The lack of stimulation of axon growth upon blockade of RhoA prompted us to investigate the role of RhoA for regenerational outgrowth of sensory neurons in more detail. Specifically, we assayed a point mutant of C3_bot_, E174Q, which lacks Rho inhibitory activity, for its potential influence on axon growth. As illustrated in Figure 5A bath application of C3_*E*174*Q*_ leads to increased total axonal outgrowth and branching. Although the overall effect was weaker than with C3_bot_, this finding was unexpected given that the Rho inhibitory activity of the mutant is virtually lost as demonstrated in Figures 5B,C. In contrast, pull down assays revealed dramatically reduced RhoA-GTP levels following 24 h of bath application of recombinant C3_bot_. In parallel, the RhoA protein was modified by ADP-ribosylation as illustrated by a shift in molecular weight (see also Just et al., 2011) or by direct labeling of RhoA with radioactive ADP in cell-free assays (Figure 5C).

### C3_bot_ stimulates ERK and Akt pathways independently from RhoA inactivation

**Figure 6:**
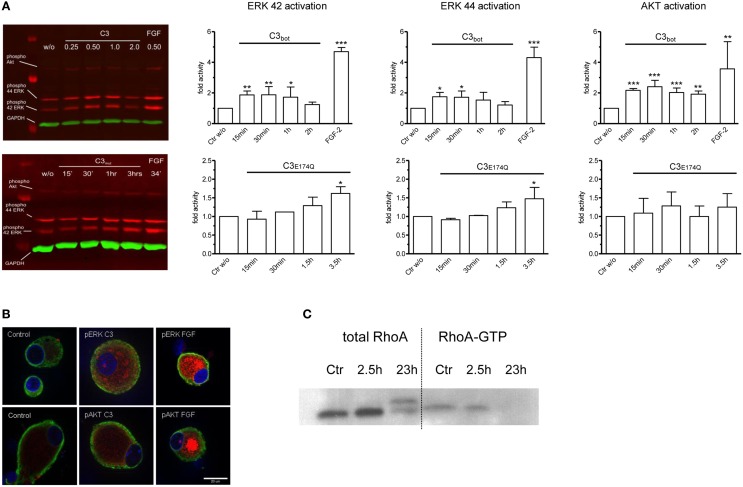
Quantitative Western blotting experiments employing infrared dye labeled secondary antibodies demonstrate rapid activation of ERK and Akt signaling pathways in dissociated adult DRG neurons after application of C3_bot_ or C3_*E*174*Q*_ (**A**; each 1 μg/ml). FGF-2 (100 ng/ml) is used as a positive control and densitometry values are normalized to GAPDH and vehicle treated control values (*n* = 3, mean ± SD; ^*^p < 0.05, ^**^p < 0.01, ^***^p < 0.005). Immunofluorescence labeling of pERK and pAkt (red) in control DRG neurons and in neurons treated with C3_bot_ or FGF-2 (**B**; 1 μg/ml for 30 min; tubulin in green and Hoechst staining in blue). Note that treatment with C3_bot_ for 2.5 h does not reduce RhoA-GTP loading suggesting a RhoA-independent activation of ERK and Akt **(C)**.

**Figure 7:**
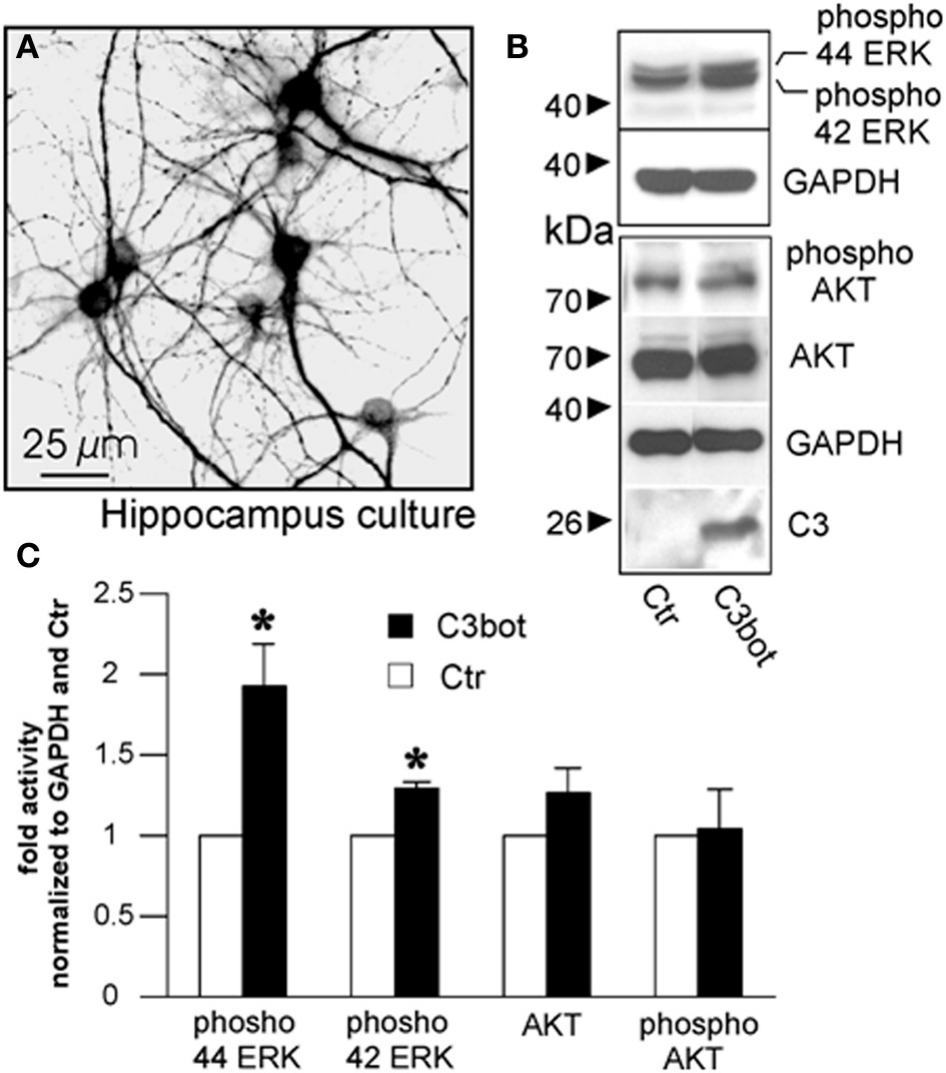
Cultured murine hippocampal neurons treated with C3_bot_ for 48 h stained for microtubule associated protein 2 **(A)** and subjected to Western blotting **(B)**. Effects of C3_bot_ treatment on ERK and Akt phosphorylation were evaluated. C3 antiserum is used to confirm presence of C3_bot_. Quantification of protein expression reveals a significant promotive effect of C3_bot_ on both ERK42 and ERK 44 phosphorylation (**C**; total number of neurons per group >50, three independent experiments, mean ± SEM; ^*^p < 0.05).

**Figure 8:**
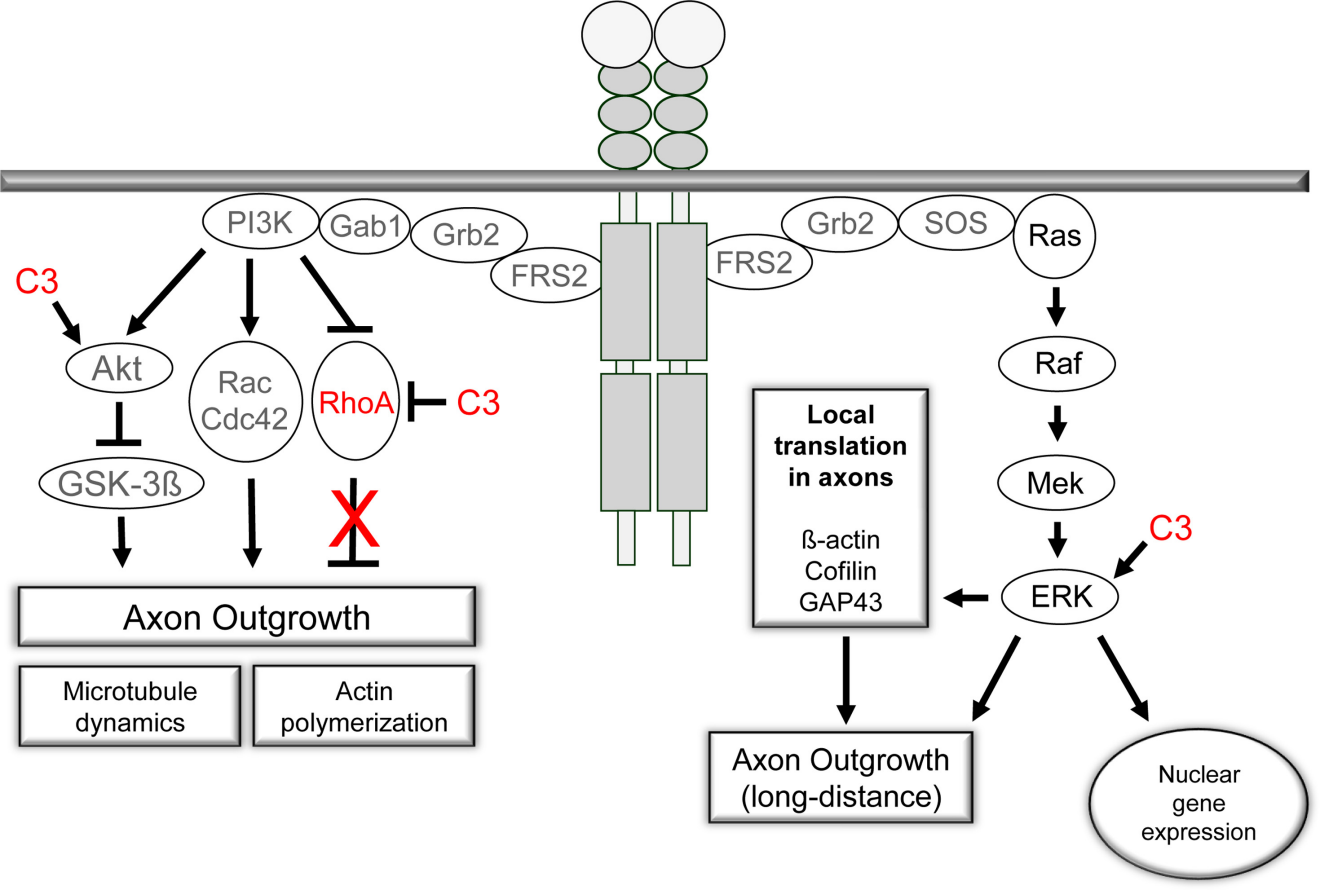
The PI3K/Akt- and Ras/Raf/ERK-signaling pathways are activated by receptor tyrosine kinases upon ligand binding. Both pathways are crucial for axon outgrowth during development and regeneration. The activation of the ERK-machinery is implicated in neuronal survival and elongative axon growth following injury. PI3K/Akt signaling is central to the regulation of cytoskeletal proteins and is linked to neuronal survival and axonal branching. Small GTPases are under control of PI3K signaling and mediate the effects on microtubule dynamics and actin polymerization. Our observations challenge the prevalent view of an inhibitory effect of RhoA activation on axonal regeneration in adult sensory neurons. Pharmacological inhibition of RhoA with C3_bot_ does not appear to underlie the positive effects of C3_bot_ on axonal regeneration. In contrast, available data suggest Rho independent means of C3_bot_ involving ERK/Akt signaling mechanisms probably by a non-enzymatic mode of action of C3_bot_.

In an approach to analyze possible signaling mechanisms underlying the Rho independent effect on axon outgrowth by C3_bot_ and C3_*E*174*Q*_, we assayed for upregulation of pERK, pAkt, and pSTAT3. All three signaling molecules are profoundly implicated in the regulation of axonal outgrowth and branching in sensory neurons (Markus et al., 2002; Zhou and Snider, 2006). We performed a C3_bot_ time course experiment and found that pERK, i.e., pERK42 and pERK44, is upregulated by about 2-fold and pAkt by about 2.5-fold within the first hour of C3_bot_ incubation with a peak at around 30 min (Figure 6A). Immunofluorescence labeling confirmed increased levels of pERK and pAkt in treated neurons (Figure 6B). pSTAT3 did not show any regulation by C3_bot_ (data not shown). C3_*E*174*Q*_, on the other hand, lead to a significant upregulation of pERK activity by about 1.6-fold after 3.5 h, but lacked any effect on pAkt. The kinetics of C3_*E*174*Q*_ affecting pERK activity were slower than of C3_bot_, which might be due to C3_bot_, but not C3_*E*174*Q*_, harboring a membrane permeable cp-tag (Winton et al., 2002). We then performed a time course assay to test whether upregulation of pERK and pAkt by C3_bot_ is dependent on its well-known Rho inhibitory activity. In contrast to cell free assays, inactivation of RhoA by C3_bot_ in DRG neurons occurs only after long-term incubation, but definitely not before 2.5 h, thus ruling out a causal link between inactivation of RhoA and activation of pERK and pAkt (Figure 6C). In order to assess the cell type specificity of this novel signaling activity of C3_bot_ we then stimulated hippocampal neurons with C3_bot_ and determined the status of pERK and pAkt. Levels of pERK, but not pAkt, were upregulated by C3_bot_ 48 h after treatment indicating some cell type specificity of C3_bot_ action, at least when comparing peripheral with central neurons (Figure 7). Shorter treatments with C3_bot_ were not effective in hippocampal neuron culture. Taken together, we suggest a model in which C3_bot_ induces kinase activation independently from inactivation of RhoA (Figure 8).

## Discussion

In the present study, we provide evidence for an axonotrophic function of C3_bot_ that is independent of RhoA inhibition in peripheral sensory neurons obtained from adult DRG. Inhibition, down-regulation or interfering with RhoA using a dominant-negative mutant did not enhance axon initiation, elongation, or branching suggesting a minor role, if any, of RhoA in axon growth of DRG neurons on a growth-promoting substrate. This outcome was surprising, since inhibition of the RhoA effector, ROCK, clearly promotes sensory axon regeneration on growth promoting or inhibitory substrates *in vitro* and *in vivo* (Borisoff et al., 2003; Fournier et al., 2003; Hiraga et al., 2006; Cheng et al., 2008). However, as summarized in a recent review (Auer et al., 2011), active RhoA influences various signal transduction pathways not only involved in negative regulation of axon regeneration, but also in stimulating axon growth, for example, via activation of mDia1 (Arakawa et al., 2003). Furthermore, cellular context and developmental stage of the neuronal model need to be considered. For example, overexpression of dominant-negative N19RhoA increased neurite extension in neuronal cell lines (Kranenburg et al., 1997; Sebok et al., 1999; Jeon et al., 2012), but decreased axon length and branching of embryonic hippocampal neurons seeded onto a growth-promoting substrate (Ahnert-Hilger et al., 2004). As we show here, this construct was ineffective in adult DRG neurons with regard to axon outgrowth. Recently, Leslie et al. deleted RhoA specifically in DRG neurons using RhoA-floxed mice under a Wnt1-Cre driver (Leslie et al., 2012). Supporting our *in vitro* data, they found that peripheral projections of sensory neurons are normal, and no detectable defects in the central projections of either cutaneous or proprioceptive sensory neurons were observed in the RhoA deficient mice. They also revealed that RhoA is not required for Sema3A-mediated axonal repulsion of sensory neurons.

The role of C3_bot_ has been extensively investigated in CNS injury models with regard to the limited regeneration due to the presence of myelin-derived inhibitors. Cerebellar granule cells treated with C3_bot_ revealed enhanced neurite outgrowth on substrate-bound axon growth inhibitors, such as Nogo-66 or MAG (Niederost et al., 2002). Moreover, sensory neurons treated with C3_bot_ did not exhibit growth cone collapse upon contact with oligodendrocytes and enhanced neurite outgrowth on myelin (Jin and Strittmatter, 1997; Fournier et al., 2003). Recently, Zhou et al. (2012) expressed C3_bot_ in cervical DRG neurons via herpes simplex virus (HSV) mediated gene transfer *in vivo*. Following unilateral dorsal root crush, large myelinated axons extending from primary afferent neurons regenerated into the dorsal columns over several segments of the spinal cord suggesting that C3_bot_ has the potential to overcome myelin-inhibition of sensory axons in the CNS. However, overexpression of full-length C3_bot_ in hippocampal (Ahnert-Hilger et al., 2004) or sensory neurons (this study) was not stimulating axonal outgrowth, whereas treatment with enzymatically inactive C3_bot_ or with peptide fragments derived from C3_bot_ promoted axon growth and regeneration in the CNS (Boato et al., 2010; Just et al., 2011).

We demonstrate here that full-length C3_bot_ rapidly induced regeneration-associated signaling pathways in primary neurons (Figure 6). The effects were weaker, but reminiscent of those induced by neurotrophic factors, such as FGF-2 or NGF, that are released upon peripheral nerve injury and enhance axon regeneration via binding to receptor tyrosine kinases followed by activation of the ERK (MAPK) and PI3K/Akt pathways (Zhou and Snider, 2006; Hausott et al., 2008). The growth factor induced decrease of RhoA-GTP levels observed in immortalized cell lines has been suggested to be relevant for these effects. NGF signaling via TrkA stimulates PI3K, which in turn increases Rac1 activity to induce RhoA inactivation (Nusser et al., 2002). In addition, the activation of FGFR1, the primary neuronal FGF receptor, and subsequent phosphorylation of the docking protein FRS2β releases Rnd1, which then decreases RhoA-GTP loading (Harada et al., 2005). Other studies performed in sensory neurons confirmed the link between the tyrosine-kinase activated subunit p110δ of PI3K and Rho inactivation (Eickholt et al., 2007). However, as demonstrated here, the inactivation of neuronal RhoA by growth factors was neither required nor sufficient for axon regeneration of adult sensory neurons *in vitro*.

The lack of axon growth promoting activities of full-length C3_bot_ in the sciatic nerve lesion model *in vivo* (Huelsenbeck et al., 2012) may be attributed to C3_bot_ mediated inhibition of RhoA in non-neuronal cells which possibly counteracted the axonotrophic effects we observed *in vitro* in a sensory neuron culture. Indeed, Schwann cell migration and axonal ensheathment are affected by perturbation of Rho activity (Sepp and Auld, 2003). In conclusion, although RhoA plays a central role in mediating the effects of myelin-derived inhibitors on axon outgrowth, its function may be limited during axon regeneration in a growth-promoting environment presented to a peripheral nerve. Nevertheless, C3_bot_ does stimulate axon outgrowth of adult sensory neurons, but not via blockade of RhoA but, presumably, via ERK and Akt activation (Figure 8). Our data presented herein support the view of a non-enzymatic mode of action of C3_bot_ (Höltje et al., 2009; Loske et al., 2012).

## Materials and methods

### Cell cultures and transfections

PC12 cells stably overexpressing fibroblast growth factor receptor (FGFR1; Hausott et al., 2008) were cultured on collagen-coated dishes in RPMI medium supplemented with 10% horse serum, 5% fetal bovine serum, antibiotics, and antimycotics (all from GIBCO Invitrogen). SH-SY5Y neuroblastoma cells were cultured in RPMI medium supplemented with 10% fetal bovine serum, 1% L-glutamine, antibiotics, and antimycotics. For transfection cells were washed off the dish, triturated for 5 min and centrifuged at 800 rpm for 5 min at 4°C. For transfection the cell pellet was resuspended in 100 μl RPMI medium and a total amount of 3 μg of plasmid was added before nucleofection (program U-029 for PC12 cells, A-023 for SH-SY5Y cells). Transfected cells were seeded onto optical dishes (ibidi) for fluorescence microscopy or onto standard plastic culture dishes for biochemical experiments.

DRG were dissected from CO_2_-euthanised young adult Sprague-Dawley rats (Charles River Laboratories). About 45 DRG per rat were collected in ice-cold RPMI supplemented with antibiotic/antimycotic and transferred into plastic dishes under a laminar flow. DRG were incubated in collagenase type I (5000 U/ml, 1 h, 37°C, GIBCO Invitrogen) followed by digestion with trypsin (0.05%, 15 min, 37°C, GIBCO Invitrogen). Two washes in 3 ml of serum containing medium (RPMI with 10% horse serum, 5% fetal bovine serum, and antibiotic/antimycotic) inhibited trypsin activity. The softened DRG were transferred to culture medium (RPMI supplemented with B27) and gently triturated 4–6 times using fire-polished Pasteur pipettes with decreasing opening diameter. The cell suspension was seeded onto plastic dishes pre-coated with poly-D-lysine hydrobromide (100 μg/ml, overnight, 37°C, Sigma-Aldrich) and laminin (5 μg/ml, >4 h, 37°C, Sigma-Aldrich).

For each transfection approximate 20 DRG were used. Following trituration, the cell suspension was centrifuged (800 rpm, 5 min, 4°C) and pellets were each dissolved in 100 μl Rat Neuron Nucleofector solution (Lonza). Five μg of plasmid DNA was added (for co-transfection experiments the fluorescent marker plasmid was applied 1:2) and suspensions were electroporated using Amaxa's nucleoporation protocol (O-003) followed by seeding the transfected neurons onto poly-D-lysine/laminin pre-coated dishes.

For preparation of hippocampal cultures neurons were prepared from fetal mice at embryonic day 16 (E16). Dissected pieces of hippocampi were rinsed with PBS, then with dissociation medium (MEM supplemented with 10% fetal calf serum, 100 IE insuline/l, 0.5 mM glutamine, 100 U/ml penicillin/streptomycin, 44 mM glucose, and 10 mM HEPES buffer) and dissociated mechanically. Sedimented cells were resuspended in starter medium (serum-free neurobasal medium supplemented with B27, 0.5 mM glutamine, 100 U/ml penicillin/streptomycin and 25 μM glutamate) and plated at a density of 1 × 10^5^ cells/well onto 6-well plates precoated with poly-L-lysine/collagen (all chemicals from GIBCO Invitrogen).

All experiments were in accordance with the statement of ethical standards concerning animal care of the guidelines of the National Institutes of Health and the ethical commission of the Austrian Ministry of Science.

### Neuron labeling and axon measurements

In experiments with low transfection efficiencies (<15%) the same DRG neuron was measured twice at 48 and 72 h after transfection. Some neurons were negatively affected by electroporation (exhibiting a reduced total axon length at day 3 when compared to day 2) and excluded from further analysis. For immunostaining, neurons were fixed for 10 min with 4% paraformaldehyde at 4°C, permeabilized with 0.5% Triton X-100 and blocked with 0.3% goat serum (in PBS) for 5 min. After rinsing in PBS primary antibodies raised against neurofilament (1:400; Sigma-Aldrich) were applied overnight at 4°C and detected by secondary antibodies conjugated to Alexa fluorochromes. For staining of hippocampal neurons a polyclonal antiserum against microtubule associated proteins 2 (MAP2, Chemicon) was used. Neurofilament positive neurons were documented by inverted fluorescence microscopy (Zeiss Axiovert 100) equipped with a SPOT RT digital camera connected to a PC. Morphometry software (MetaMorph®, Visitron Systems) was applied to measure the longest of all vectors from the centroid of the cell body to the growth cones (the maximal axonal distance, MD), the total axonal length and the number of all axonal branch points per neuron. All morphologically intact neurons per dish with MDs ≥ 50 μm were documented by high-resolution CCD cameras followed by image correction for background and cell body fluorescence.

### Plasmids and C3_bot_ preparations

pEGF-C3_bot_ was a kind gift of K. Kosonen and M. Courtney (Kuopio, Finland). Plasmids encoding dominant negative, constitutively active and wild type RhoA have been described before (Ahnert-Hilger et al., 2004). For down-regulation of RhoA shRNA plasmid (pCMV-EGFP-U6-rRhoA618) was used (top strand of the insert 5′ to 3′ : GAT CCG TGC TGT TTA TTA ATC TTA TTC AAG AGA TAA GAT TAA TAA ACA GCA CTT TTT TGA ATT CA). *Clostridium botulinum* derived mutant C3_bot_ (C3 _*E*174*Q*_, carrying a point mutation from glutamate to glutamine at AA 174) was cloned into pGEX-2T expression vectors and expressed as recombinant glutathione *S*-transferase fusion protein in *Escherichia coli* TG1. The GST tag was removed by thrombin cleavage. C3_bot_ (membrane permeable) was purchased from Cytoskeleton.

### RT-PCR

RNA extraction was performed applying the RNeasy Mini Kit (Qiagen) including DNase-digestion. The transcription of RNA to cDNA was carried out with the iScriptTM cDNA Synthesis Kit (Biorad) according to the manufacturer's instructions with 5 min at 25°C, 30 min at 42°C, and 5 min at 85°C resulting in 20 μl of cDNA. Quantitative real time (qRT) PCR of cDNA from cultured primary sensory rat DRG neurons or PC12 cells was performed on Biorad's iCycler in a final volume of 25 μl with 12.5 μl iQ SYBR Green Supermix (Biorad; containing 40 mM Tris-HCl, 100 mM KCl, 6 mM MgCl_2_, 0.4 mM of each dNTP, 50 U/ml iTaq DNA polymerase, SYBR Green I, 20 nM fluorescein, and stabilizers), 7.5 μl sterile water, 0.2 μl of each primer (20 μM), and 1 μl cDNA template (3 min denaturing step followed by 40 cycles 30 s at 95°C and 60 s at 60°C). The following primers were used: hypoxanthine phosphoribosyltransferase 1 (HPRT1) as a house keeping gene control (forward TGACACTGGCAAAACAATGCA and reverse GGTCCTTTTCACCAGCAAGCT), C3 (forward AGCAAAAGGCTCAAAGGCAGGAT and reverse GCTGTGCCCATCATTGTTGCTGT), RhoA (forward CTCATAGTCTTCAGCAAGGACCAGTT and reverse ATCATTCCGAAGATCCTTCTTGTT).

The verification of PCR products was performed with both melt-curve analysis (Biorad iCycler iQ software) and electrophoretic separation on a 2% agarose gel containing 0.1% ethidium bromide with subsequent visualization of DNA bands by ultraviolet transillumination.

Grouped cell qRT-PCR was based on 20 cells per group. Micromanipulator-supported microscopy was used to pick individual single cells under visual control. Cells were sucked into a glass capillary with a diameter of approximately 20 μm, and released into lysis/binding buffer (DYNAL Dynabeads mRNA direct micro kit). The mRNA was isolated by magnetic separation on 10 μl prewashed magnetic beads, washed, eluted in TrisHCl and solid-phase cDNA synthesis was run according to the manufacturers protocol, using 4 μl reaction mix, 1 μl reverse transcriptase, and 10 μl nuclease-free water per sample (iSCRIPT Biorad). Five μl of cDNA and 12.5 μl of iQ SYBR Green Supermix (Biorad) were diluted with destilled water to a final volume of 25 μl and administered to qPCR. The applied steps of the polymerase chain reaction and primers in use are stated above.

### Western blotting

For Western blotting experiments cells were washed twice in ice cold PBS, scraped, and lysed in RIPA II lysis buffer consisting of 50 mM Tris/HCl pH 7.4, 500 mM NaCl, 1% NP-40, 0.5% Na-DOC, 0.1% SDS, 0.05% NaN3, protease inhibitor cocktail Complete (1:100, Roche Applied Science) and phosphatase inhibitor cocktails (1:100, Sigma). Lysates were incubated on ice for 45 min and vortexed every 10 min, sonicated and centrifuged (14,000 rpm, 20 min, 4°C) in order to collect the supernatant. As a positive control 0.1 μg of C3_bot_ was used. Homogenates dissolved in 4× Laemmli buffer (0.25 M Tris pH 6.8, 8% SDS, 40% glycerol, bromphenol blue, 20% β-mercaptoethanol) were denaturated at 95°C for 5 min, loaded on 10% or 15% polyacrylamide gels, separated via electrophoresis at 100V, and blotted onto polyvinylidene difluoride (PVDF) membranes. These were blocked with 5% skim milk (Merck) and incubated overnight at 4°C with antibodies against RhoA (1:1000, Santa Cruz) in 1% skim milk in PBS containing 0.05% Tween20 (PBS-Tween). Antibodies (all from Cell Signaling) against pAkt (1:2000), pERK (1:1000), pSTAT3 (1:1000), Akt (1:2000) or ERK (1:2000) were applied in PBS-Tween containing 5% BSA, antibodies against C3 (1:5000; Hoffmann et al., 2008) in PBS-Tween containing 2% milk and 3% BSA. Gross protein load was assessed using Ponceau red, while GAPDH bands served as loading controls (1:1000; Santa Cruz). Secondary horseradish peroxidase-linked anti rabbit or anti mouse antibody (Pierce; 1:10,000) was detected by the enhanced chemiluminescence Western blotting detection system (Pierce). Quantitative Western Blotting was carried out using secondary IR 800 antibody (anti mouse) and IR 680 antibody (anti rabbit) from LI-COR, both applied at 1:2500 in 5% BSA, and detected with an Odyssey® Infrared Imaging System (LI-COR). Quantification of signal intensities was performed with the Odyssey® software.

### RhoA-GTP pulldown

Approximately 45 DRG were collected per sample. Dissociated neurons were cultured for 24 h with or without treatment of C3 enzyme as indicated, followed by protein extraction at 4°C as described (Schweigreiter et al., 2004). Briefly, cells were washed twice with ice-cold TBS and lysed with ice-cold lysisbuffer [500 μl per sample; 50 mM Tris pH 7.2, 1% Triton X-100, 150 mM NaCl, 10 mM MgCl_2_, plus freshly added protease inhibitor cocktail Complete (Roche; 1:100)]. Lysed cells were scraped, collected into a pre-cooled Eppendorf tube and centrifuged at 14,000 rpm at 4°C for 5 min. Supernatants were divided into a major portion dedicated to the pull-down of RhoA-GTP (about 450 μl) and a minor portion of 50 μl for assessment of total RhoA. RhoA-GTP was affinity precipitated by incubating with several microgram of immobilized GST-RBD for 50 min at 4°C. GST-RBD beads were washed three times (700 ×g 1 min, 4°C, 500 μl lysis buffer) and elution and denaturation was done with 1× Laemmli buffer at 95°C for 5 min. Samples were electrophoresed in 15% polyacrylamide and proteins were transferred onto PVDF membranes, which were blocked in 1% skim milk solution and incubated with anti-RhoA antibody (1:1000).

### Cell-free ADP ribosylation assay

Cells were washed with PBS and scraped into 100 μl of lysis buffer (50 mM Tris-Cl, pH 7.4, 1% Triton, 10 mM NaCl, 5 mM MgCl_2_, 1 mM PMSF, 5 mM DTT) followed by ultrasonic disruption on ice. Protein concentrations were measured by the Bradford method. Cell lysates containing equal amounts of protein were incubated with recombinant exoenzyme (C3_bot_ or C3_*E*174*Q*_) and 1 μ Ci [^32^P]NAD (Amersham Life Science), 10 mM dithiothreitol, 10 mM thymidine, and 5 μM NAD at 37°C for 20 min. The reaction was terminated by addition of Laemmli sample puffer and then incubated at 95°C for 10 min. Samples were resolved by SDS-PAGE on 15% gels, and the ADP-ribosylated Rho was analyzed by phosphorImaging.

### Statistics

Values are expressed as means ± SD or SEM (as indicated in figure legend). Each experiment was performed at least three times. Graph Pad Prism 5 software was used to perform graphics and to analyze statistical significance (Student's *t*-test, One-Way or Two-Way ANOVA with Tukey post-test if appropriate). *P*-values below 0.05 were considered statistically significant.

### Conflict of interest statement

The authors declare that the research was conducted in the absence of any commercial or financial relationships that could be construed as a potential conflict of interest.
